# Dexamethasone Versus Placebo in Comatose Survivors of Out‐of‐Hospital Cardiac Arrest: Study Protocol for a Multicenter Randomized Comparison Within the Danish Out‐of‐Hospital Cardiac Arrest Trial (DANOHCA)

**DOI:** 10.1111/aas.70287

**Published:** 2026-06-24

**Authors:** Jo B. Andreasen, Anders Morten Grejs, Simon Mölström, Lars P. K. Andersen, Sören Möller, Steffen Christensen, Henrik Schmidt, Jacob E. Møller, Martin A. S. Meyer, Jesper Kjærgaard, Alexander W. Erbs, Bodil S. Rasmussen, Christian Hassager

**Affiliations:** ^1^ Department of Anaesthesiology and Intensive Care Aalborg University Hospital Gistrup Denmark; ^2^ Department of Clinical Medicine, Faculty of Medicine Aalborg University Aalborg Denmark; ^3^ Department of Intensive Care Aarhus University Hospital Aarhus Denmark; ^4^ Department of Clinical Medicine, Faculty of Health Aarhus University Aarhus Denmark; ^5^ Department of Anaesthesiology and Intensive Care Odense University Hospital Odense Denmark; ^6^ Department of Clinical Research, Faculty of Health Sciences University of Southern Denmark Odense Denmark; ^7^ Department of Cardiothoracic Anaesthesiology Copenhagen University Hospital—Rigshospitalet Copenhagen Denmark; ^8^ Department of Intensive Care Zealand University Hospital, Koege Hospital Køge Denmark; ^9^ Department of Clinical Medicine, Faculty of Health Sciences University of Copenhagen Copenhagen Denmark; ^10^ Epidemiology, Biostatistics and Biodemography, Department of Public Health University of Southern Denmark Odense Denmark; ^11^ Department of Cardiology, the Heart Centre Copenhagen University Hospital—Rigshospitalet Copenhagen Denmark; ^12^ Department of Cardiology Odense University Hospital Odense Denmark

**Keywords:** critical care, dexamethasone, out‐of‐hospital cardiac arrest, post‐resuscitation care, randomized clinical trial

## Abstract

**Background:**

The inflammatory response to whole‐body ischemia, as seen in comatose survivors after out‐of‐hospital cardiac arrest (OHCA), is associated with increased morbidity and mortality. Early high‐dose dexamethasone may dampen the inflammatory response, which may diminish secondary brain damage and other organ injury and decrease mortality. The aim is to evaluate the effect of high‐dose dexamethasone in post‐resuscitation care.

**Methods:**

The Danish Out‐of‐Hospital Cardiac Arrest (DANOHCA) trial is an investigator‐initiated, multicenter, randomized, controlled trial evaluating four post‐resuscitation interventions in adult patients resuscitated after OHCA including blinded early administration of high‐dose dexamethasone compared with placebo (isotonic saline). Adult OHCA patients with presumed cardiac etiology and sustained return of spontaneous circulation will be randomized 1:1 to receive 20 mg of dexamethasone or placebo immediately after inclusion in the trial and repeated the following two mornings for a total of three doses. The primary outcome is all‐cause mortality within 90 days using a log‐binomial regression analysis to estimate the relative risk of death from any cause between randomization and Day 90. Enrollment began in June 2023, with the aim to include 1000 patients at five sites in 3–4 years. The trial is registered at clinicaltrials.gov (identifier: NCT05895838) and euclinicaltrials.eu (2024‐515997‐28‐00).

**Perspectives:**

It is of paramount importance to optimize post‐resuscitation care through large‐scale randomized trials. Global ischemia–reperfusion–induced inflammatory response with resulting organ failures represents a possible target for interventions after OHCA. This part of the DANOHCA trial will provide new evidence on the impact of high‐dose dexamethasone in survivors of OHCA.

**Trial Registration:** Clinical Trials Information System (CTIS): 2024‐515997‐28‐00

## 1 Introduction

Out‐of‐hospital cardiac arrest (OHCA) remains a major cause of death and disability worldwide despite advances in prehospital logistics, bystander‐performed basic life support, cardiopulmonary resuscitation (CPR) techniques, and post‐resuscitation care [1, 2]. Those who are successfully resuscitated and admitted to intensive care unit (ICU) in a comatose state have a high risk of dying either from multi‐organ failure or from severe hypoxic–ischaemic brain injury and subsequent withdrawal of life‐sustaining therapies [3]. The clinical course after OHCA and whole‐body ischemia is commonly characterized by various degrees of brain injury, myocardial dysfunction, global ischemia–reperfusion injury, and persistent precipitating pathology, named the post‐cardiac arrest syndrome (PCAS) [4, 5]. The systemic inflammatory response induced by ischemia and reperfusion injury progresses during the first 1–3 days following OHCA and is associated with poor outcome [6].

### 1.1 Glucocorticoids to Dampen Inflammatory Response

Inflammatory markers associated with poor outcome after OHCA include interleukin (IL)‐6, high sensitivity C‐reactive protein (hsCRP), leukocytes, IL‐1b, IL‐10, IL‐13, tumor necrosis factor alpha (TNF‐alpha), and procalcitonin [7]. IL‐6 receptor antagonists have been shown to modulate the systemic inflammation with a decrease in markers of myocardial injury in comatose OHCA patients [8]. Anti‐inflammatory treatment with glucocorticoids may reduce the severity of PCAS by suppressing the global ischemia–reperfusion–induced inflammatory response and stabilizing endothelial function [9]. In addition, glucocorticoids enhance vasopressor responsiveness due to relative adrenal insufficiency and contribute to improved hemodynamics after ROSC [10, 11]. The recent Vasopressin and Methylprednisolone for In‐Hospital Cardiac Arrest (VAM‐IHCA) trial demonstrated a significant improvement in return of spontaneous circulation (ROSC) as compared to placebo but no clear effect on long‐term outcomes (survival nor neurological outcome) [12]. The STEROHCA trial [13] compared prehospital administration of methylprednisolone versus placebo for OHCA and found significantly reduced IL‐6 levels in the intervention group but no difference in neuron‐specific enolase (NSE) up to 72 h after cardiac arrest. No significant difference in 180 days mortality was found between the two groups evaluated by the crude hazard ratio (HR). However, a significant difference in favor of the intervention was found in a multivariate model adjusting for sex, age, primary rhythm, time to ROSC, and primary percutaneous coronary intervention. Also, a sub‐study found a significant reduction in a panel of inflammatory markers at 24 h in the intervention group compared to the placebo group [14].

As confirmed in two recent systematic reviews and meta‐analyses [15, 16], uncertainty remains regarding the benefits of glucocorticoids as an anti‐inflammatory treatment to dampen the inflammatory response triggered by whole‐body ischemia and reperfusion injury in OHCA survivors. We hypothesize that high‐dose dexamethasone reduces 90‐day all‐cause mortality in comatose OHCA survivors compared to placebo by dampening the inflammatory response and reducing organ failures caused by ischemia and reperfusion injury.

### 1.2 Objective

This part of the ongoing nationwide Danish OHCA (DANOCHA) trial aims to evaluate the impact of high‐dose dexamethasone on 90 days mortality as one of four interventions (full protocol: www.danohca.dk). Here, we describe the protocol for the dexamethasone versus placebo intervention in detail.

## 2 Methods

### 2.1 Study Design

The DANOHCA trial is an investigator‐initiated, randomized, controlled, multicenter clinical trial with co‐enrolment into four different interventions, that is, 5° versus 35° backrest elevation, early (≤ 6 h) versus late (28–36 h) wake‐up call, dexamethasone versus placebo, and olanzapine versus placebo. Intervention‐specific rationale and procedures are described in four corresponding protocol manuscripts (LINK; Olanzapine, WUC, Headrest) and a statistical analysis plan that outlines the methodological approach to analyzing the common primary, secondary, and tertiary outcomes of the DANOHCA trial (LINK: SAP). After completion of screening procedures, patients are randomized in a 1:1 fashion to receive either the active intervention or the placebo/control intervention for each of the four interventions. The randomization sequence was developed using the PROC PLAN procedure in SAS (SAS Institute Inc., Cary, NC, USA). The randomization is revealed by opening a sealed box containing allocation to backrest positioning, time for wake‐up call, and blinded vials/tablets for dexamethasone and olanzapine, respectively, as well as a unique trial identifier and a QR code for data entry into the electronic case report form (eCRF). The study is not stratified by trial site as sealed boxes are prepared centrally and delivered to trial sites randomly based upon the inclusion rate.

The DANOHCA trial is registered at clinicaltrials.gov (identifier: NCT05895838) and at the EU CTIS under 2024‐515997‐28‐00 accessible at euclincaltrials.eu (previously registered with EudraCT number: 2021‐005876‐21 at clinicaltrialsregister.eu). The trial has been approved by the Danish Medicines Agency (DKMA) and Danish Medical Research Ethics Committees in accordance with the Clinical Trials Regulation No 536/2014 and registered with CTIS trial number 2024‐515997‐28‐00; transition authorized with conditions on 11‐10‐2024; the current protocol v02‐12‐2025 (SM‐2) was authorized on 05‐12‐2025. Prior to the transition to CTR the trial had been approved by the DKMA no. 2021121966 since 07‐02‐2022 and the regional ethics committee of the Capital Region journal number H‐21077461 since 08‐07‐2022.

### 2.2 Trial Conduct

The trial is conducted in accordance with the applicable national and international guidelines including the revised Declaration of Helsinki [17], and the International Good Clinical Practice guidelines [18]. The trial and the protocol adhere to the International Conference on Harmonization (ICH) guidelines [1, 2, 3, 19, 20] and the Standard Protocol Items: Recommendations for Interventional Trials (SPIRIT) statement [21, 22]. The trial is approved as emergency research with proxy consent, as the interventions should be started as soon as possible, and the patients included are incapacitated. The informed consent is obtained from an appointed legal representative, that is, a physician independent of the trial, and thereafter from next of kin as soon as possible. Informed consent from the patient is obtained as soon as possible when the patient regains capacity according to the Danish legislation and the European Clinical Trial Regulation [23, 24, 25, 26]. Screening is performed as soon as possible after hospital admission by the physician in charge. The screening log includes all patients admitted to ICU after OHCA with inclusion of the patient's ID, demographic data, and reasons for the exclusion of screened patients (lack of inclusion criteria, presence of exclusion criteria, or logistical reasons).

DANOCHA is a pragmatic trial, and all other interventions/treatments are at the discretion of the treating physicians with adherence to international and national guidelines for post‐resuscitation care [3, 27].

### 2.3 Trial Intervention

Upon arrival at the ICU, patients are allocated by 1:1 randomization to receive either 20 mg dexamethasone (Dexavit, Vital Pharma Nordic ApS, Denmark) or placebo (0.9% saline). The study drug or placebo is administered intravenously (iv) over 15 min at randomization, followed by 20 mg dexamethasone or placebo iv daily at 06:00 am or at least 8 h after the initial dose for 2 days with a total of three doses. Concurrently, a 1:1 randomization is performed for each of the three other interventions in the DANOHCA trial.

### 2.4 Blinding

The dexamethasone versus placebo intervention is blinded to both clinical personnel, site investigators and statisticians. Identical‐looking vials with identical color of the fluid and identical volumes of the study drug (dexamethasone) and placebo (saline 0.9%) are produced by Vital Pharma Nordic ApS, Denmark and The Hospital Pharmacy of the Capital Region, respectively. The drug administration schedule is identical for patients receiving active medication and placebo and registered both in medical records and study eCRF for monitoring of adherence. During the conduction of the trial, the concealment will only be broken in the case of a serious adverse event (SAE) or a suspected unexpected SAE at the trial sponsor's discretion.

### 2.5 Patient Eligibility and Enrollment

The trial is led and coordinated by the Department of Cardiac Intensive Care, Copenhagen University Hospital—Rigshospitalet, Copenhagen, Denmark. Comatose resuscitated OHCA patients with suspected cardiac etiology are enrolled consecutively at five university hospitals covering the entire Danish population, that is, Copenhagen University Hospital—Rigshospitalet, Aalborg University Hospital, Aarhus University Hospital, Odense University Hospital, and Zealand University Hospital—Koege.

All OHCA patients admitted to one of the five participating ICUs are screened for inclusion. Eligible OHCA patients fulfilling all inclusion criteria and no exclusion criteria (see Table 1) are included as soon as possible and no later than 3 h after ROSC. As soon as possible after inclusion in the trial, a sealed box containing trial medication and allocation group for the two physical interventions is opened, and the four interventions are initiated. Co‐enrollment in other trials is considered individually, according to the principles of the SPICE‐8 statement [28], and must be accepted by the steering committee following careful evaluation of the risk of interaction with the four per‐protocol interventions.

**TABLE 1:** Inclusion and exclusion criteria.

Inclusion criteria	Exclusion criteria
All the listed criteria (1–4.) must be met. 1. Age ≥ 18 years 2. OHCA of presumed cardiac cause. 3. Sustained ROSC.^a^ 4. Unconsciousness (GCS < 9) (patients not able to obey verbal commands) after sustained ROSC at the time of randomization	Females of childbearing potential if pregnancy is suspected (unless a negative HCG test can rule out pregnancy within the inclusion window). Known bleeding diathesis (medically induced coagulopathy (e.g., warfarin, NOAC, clopidogrel) does not exclude the patient). Suspected or confirmed acute intracranial bleeding. Suspected or confirmed acute stroke. Unwitnessed asystole. Known limitations in therapy and Do Not Resuscitate‐order. Known disease making 180 days survival unlikely. Known pre‐arrest Cerebral Performance Category (CPC) 3 or 4 functional status. > 3 h (180 min) from ROSC to screening. Systolic blood pressure < 80 mmHg despite fluid loading/vasopressor and/or inotropic medication.^b^ Use of intra‐aortic balloon pump/axial flow device/ECMO.^c^ Temperature on admission < 30°C. Known allergy for dexamethasone or olanzapine. Ongoing (within 48 h) treatment with olanzapine or dexamethasone. Known back or hip condition that precluded the patients from being positioned with backrest from 0 to 45‐degree angle. Known or suspected long QT syndrome (LQTS) Known active fungal disease. Localized skin lesions do not exclude patients from inclusion. Estimated body weight < 45 kg.

*Note:* Since post resuscitation care and intervention are regarded as emergency procedures and should not be delayed, there should only be taken easily accessible sources (previous in‐house chart records, history from emergency medical services) to assess criteria 2, 5, 6, 7, 8, 12, 13, 14, and 15.

^a^ Sustained ROSC is when chest compressions or mechanical circulatory support have been not required for 20 consecutive minutes and signs of circulation persist.

^b^ If the systolic blood pressure (SBP) is recovering during the inclusion window (180 min), the patient may be included.

^c^ If the patient is weaned and the device is removed during the inclusion window (180 min), the patient may be included.

### 2.6 Endpoints

#### 2.6.1 Primary Endpoint

All‐cause mortality at 90 days after OHCA.

#### 2.6.2 Secondary Endpoints

- Number of patients with one or more serious adverse reactions (SARs) to dexamethasone in the ICU.
- Days alive and out of hospital at 30 days (actual number of days, calculated by subtracting the time spent in the hospital or deceased within 30 days, then rounding down to the nearest whole number).
- Cerebral Performance Category (CPC) and Modified Rankin Scale (mRS) at follow‐up 3–6 months after cardiac arrest (conducted either through physical attendance or telephone interview according to participant preference).
- Serum neuron specific enolase (NSE) at 48 and72 h.
- Markers of inflammation; hsCRP. platelet‐, leukocyte‐ and differential count daily during the initial 72 h.

#### 2.6.3 Explorative Endpoints

- Days in need of vasopressors within 30 days.
- Duration (days) of invasive mechanical ventilation within 30 days
- Days with renal replacement therapy (RRT) within 30 days
- Assessment of organ failure other than cerebral during ICU stay; troponin‐I, troponin‐T, creatine kinase‐MB, alanine transaminase, bilirubin, albumin, and creatinine.
- Visco‐elastic coagulation profile, plasmatic coagulation profile, and markers of endothelial activation, as well as inflammatory response daily during ICU stay up to 72 h

### 2.7 Statistical Methods

#### 2.7.1 General Principles

The four interventions in the DANOHCA trial are considered as four individual trials and data will be analyzed and reported individually, hence no adjustment for multiple comparison is planned for the primary endpoint. A full statistical analysis plan is published together with the four separate protocol articles (LINK: SAP). Since previous trials in patients after OHCA predominantly yielded neutral results, all four interventions are accepted without consideration of potential interactions. If more than one intervention demonstrates a non‐neutral effect, a post hoc evaluation of possible interactions and potential safety aspects will be conducted and reported. Also, as an investigation of potential safety aspects related to possible interactions between the four interventions, we will report on interactions related to mortality and occurrence of other SAEs.

All analyses will be performed in the modified intention‐to‐treat (ITT) population, applying a two‐sided significance level of 0.05, as an interim analysis has been performed after inclusion of 500 patients. The modified ITT population is defined as patients having received all scheduled doses of dexamethasone, with exclusion of any patients erroneously enrolled.

#### 2.7.2 Sample Size Considerations

We estimate a total mortality of 35% within 90 days and aim for 25% relative risk reduction (to a mortality of 26%). With a power of 85% and an *α* of 5%, a sample size of 468 patients in each group is needed. To account for dropouts and loss to follow‐up, albeit expected to be minimal, we plan to include 500 patients in each treatment arm, for a total of 1000 patients.

#### 2.7.3 Analysis of Survival Data (Primary Outcome)

The primary analysis will use log‐binomial regression to estimate the relative risk of 90‐day mortality, defined as the proportion of patients who die from any cause between randomization and day 90. Kaplan–Meier curves for each allocation group will be estimated, graphically displayed, and compared by the log‐rank test. Further Cox proportional hazard models will be applied to assess differences in time to death between treatment groups, estimating HRs with 95% confidence intervals. These models will sequentially be adjusted for interaction between treatment allocations, and each of the following variables: sex, age, time to ROSC, lactate level upon admission, shockable primary rhythm, witnessed cardiac arrest, bystander performed CPR, presence of STEMI in the admission ECG. These interactions will be reported in a forest plot. Further, models considering cause of death (cardiovascular, neurological, multi‐organ failure, other) as competing risks will be performed by estimating the cumulative incidence for each type of death by the Nelson–Aalen estimator. Any changes from the pre‐specified analysis plan will be reported.

#### 2.7.4 Supplementary Analyses

Throughout, categorical variables will be presented as counts with proportions, whereas continuous variables will be presented as mean ± SD if normally distributed, and as median (25th percentile–75th percentile) if non‐normally distributed, as evaluated by quantile‐quantile plots. Categorical endpoints by treatment allocation will be analyzed by the chi‐squared or Fisher's exact test, as appropriate. Continuous endpoints will be analyzed by application of Wilcoxon ranksum test and reported with median differences with 95% confidence intervals (non‐parametric bootstrapping with 1000 resamples in case of deviations from distributional assumptions).

#### 2.7.5 Missing Data

Missing data for the primary outcome is expected to be minimal (< 5%) given the registry‐based follow‐up for mortality. The primary analysis will include all participants with available data. For secondary and tertiary outcomes, missing data mechanisms will be evaluated, and multiple imputations will be used if appropriate.

### 2.8 Sub‐Studies

Predefined sub‐studies will describe the inflammatory response in terms of cytokines and other mediators after OHCA and evaluate the effect of dexamethasone on these as well as the clinical implications of eventual findings. Also, we will compare visco‐elastic coagulation measures in the two groups, conventional coagulation parameters, and biomarkers of inflammation and endothelial activation to investigate if ischemia–reperfusion–induced inflammatory response, endothelial activation, and coagulation activation are closely related and may be attenuated by treatment with high‐dose dexamethasone.

### 2.9 Biobank

A biobank containing blood drawn upon arrival in ICU and daily up to 72 h has been established. This will provide the basis for important predefined sub‐studies on glucocorticoid effect after OHCA as well as potential new insight into the pathophysiology behind PCAS.

### 2.10 Publication Plan

The findings from each of the four DANOHCA interventions will be reported as separate manuscripts submitted to peer‐reviewed international journals, and all results—whether beneficial, neutral, or harmful—will be published. In accordance with the EU Clinical Trials Regulation, summary results will also be publicly accessible through the EU Clinical Trials Information System (CTIS) and automatically posted on the EU Clinical Trials Register within 1 year of the end of the trial. The statistical analysis plan will be published before the last patient is included in the trial.

### 2.11 Safety and Adverse Events (AEs)

Potential risks associated with high‐dose dexamethasone are possible cardiovascular side effects in the form of bradycardia and arrhythmias associated with the infusion of pulse doses of glucocorticoids if given within a short period. The Danish Summary of Product Characteristics recommends that the initial dose of dexamethasone be administered over at least 5 min. We therefore consider it safe and potentially beneficial to administer dexamethasone iv over a period of 15 min for all three doses given in the trial.

#### 2.11.1 AEs

Comatose OHCA patients requiring post‐resuscitation intensive care have a high risk of morbidity and mortality and thus, a high probability of AE. Therefore, the following events will not be considered an AE in the present study, as they are frequently occurring in OHCA patients during ICU stay: myocardial infarction, minor bleeding, metabolic disorder, and other biochemical abnormalities.

AEs are categorized according to the following definitions: major bleeding, infections (pneumonia, sepsis, pyelonephritis, upper airway infections), renal impairment requiring RRT, electrolyte disorders, metabolic disorders (hypoglycemia, prolonged hyperglycemia), cardiac events (ventricular fibrillation, ventricular tachycardia, atrial fibrillation requiring cardioversion, new need for pacing, prolonged QT interval > 560 ms, circulatory collapse), respiratory events (self‐extubation, need for reintubation), seizures (tonic–clonic, myoclonic, status epilepticus), extrapyramidal side effects, skin complications, and death after withdrawal of life‐sustaining therapy. The following AEs are considered potentially related to the dexamethasone intervention (adverse reactions, ARs): infections (pneumonia, sepsis, pyelonephritis, upper airway infections), hyperglycemia, skin complications (acne, atrophy, purpura, reduced wound healing). For each AE reporting, there will be an additional question regarding whether there has been a SAE/SAR, defined as an AE/AR that results in death, is life threatening, requires prolongation of hospitalization, or results in significant disability. Suspected unexpected serious adverse reactions (SUSARs) will be reported by the sponsor to the EudraVigilance database as soon as possible and no later than 7 days after registration.

#### 2.11.2 Monitoring of AEs and SAEs

Patients are monitored for the development of an AE for 30 days after randomization or until hospital discharge if this occurs later than 30 days. A pre‐specified form in the electronic CRF allows for daily recording of AE during ICU stay. AE occurring after ICU discharge and within 30 days from randomization will be evaluated at the time of ambulatory follow‐up (scheduled for 3–6 months after cardiac arrest). AE will only be reported to the trial sponsor if they can be classified as a SAE with a suspected direct relation to the trial intervention. Once a year, all SAEs occurring at the trial centers will be submitted and a safety report of all trial patients will be submitted to the competent authorities as required. All SAEs will be reported as endpoint measures in the final trial report.

#### 2.11.3 Interim Analysis

An Independent Data Monitoring Committee (IDMC) consisting of three members with expertise in cardiac arrest research and intensive care was appointed before the start of the trial and is responsible for safeguarding the interests of trial participants, assessing the safety and efficacy of the interventions during the trial, and monitoring the overall conduct of the clinical trial following a charter agreed upon before trial commencement. An interim analysis with de‐identified data for safety based on the respective primary endpoints for the four interventions and the occurrence of the protocol‐specified categories of SAEs was performed after 500 patients out of the preplanned 1000 patients. The interim analysis was performed as a safety review without formal efficacy or futility testing by the IDMC, resulting in no remarks.

### 2.12 Trial Status and Timeline

Randomization began in June 2023, and patient inclusion is currently proceeding according to the expected timeline, with the last patients of a total of 1000 patients expected to be included in July 2026. Follow‐up is expected to be completed in October 2026.

## 3 Discussion

The DANOHCA trial will evaluate four commonly used and potentially beneficial interventions in comatose OHCA patients admitted to the ICU. The evidence regarding each of the interventions is sparse, and the purpose of the trial is to evaluate whether these four interventions will minimize the morbidity and mortality associated with PCAS, including the impact of early administered high doses of dexamethasone. Despite PCAS being associated with a high inflammatory burden, there is a lack of effective treatment addressing this. Several studies have shown potential for glucocorticoids as treatment, but the clinical effects remain uncertain. This large‐scale randomized clinical trial is designed to address this important knowledge gap and is powered to detect a clinically meaningful difference in 90‐day all‐cause mortality among patients with OHCA.

### 3.1 Strengths

This trial has several strengths. It is a nationwide, investigator‐initiated, multicenter trial testing commonly used treatments, all with conflicting or limited evidence. The trial has a pragmatic design, making it feasible and easy to embed in routine clinical practice and is conducted in all cardiac centers representing each of the five Danish Regions with similar monitoring infrastructure. The dexamethasone versus placebo intervention is blinded to both clinical personnel, site investigators, and statisticians, which minimizes the risk of bias. The risk of side effects and complications is likely to be limited, as treatment time is short and dexamethasone is a registered, well‐known drug given within normal dosage regimens. Patient‐centered outcomes capture both survival and degrees of PACS.

### 3.2 Limitations

As only Danish centers participate in the study, global external validity may be limited to countries providing similar standards of care after resuscitation. Also, the DANOHCA trial is not powered to evaluate interactions between the four interventions but allows analysis for each intervention with preserved statistical validity of the primary hypothesis within each domain. Although exploratory cross‐arm interaction analyses are planned—mainly to assess potential synergistic or antagonistic effects—they are acknowledged as hypothesis‐generating only.

## 4 Conclusion

The DANOCHA trial will evaluate interventions to improve outcomes after OHCA using the highest standard of clinical research. We will evaluate the effect of early high‐dose dexamethasone versus placebo on outcome in resuscitated comatose OHCA patients admitted to the ICU for life support. This large trial will generate robust knowledge to potentially inform future clinical practice and provide the basis for future studies conducted with the highest standard of further clinically relevant treatments.

## Author Contributions

Jesper Kjaergaard, Jacob E. Möller, Henrick. Schmidt, Jo B. Andreasen, Bodil S. Rasmussen, Anders Morten Grejs, Steffen Christensen, Lars P.K. Andersen, Simon Mölström, Martin A.S. Meyer, and Christian Hassager designed the study protocol of the DANOHCA trial. Together with Sören Möller these authors wrote the statistical analysis plan. Jo B. Andreasen and Bodil S. Rasmussen wrote the first draft of the manuscript. All authors contributed to manuscript editing and approved the final version of the manuscript.

## Funding

The DANOHCA trial has applied for funding by external foundations for medical research and has received an unrestricted research grant of 14.8 million DKK from the Novo Nordisk Foundation (NNF22OC0079649), and 3.2 million DKK from the Danish Heart Foundation (grant number: 2023‐12401); neither the Novo Nordisk Foundation nor the Danish Heart Foundation has a financial interest in the study or participated in the design or execution of the trial.

## Conflicts of Interest

Jesper Kjaergaard reported research grants from the NovoNordisk Foundation NNF22OC0079649 (current trial) and Sundhedsdonationer 2025‐0271 (outside the current trial). Speaker's honorarium from Vicare A/S C. Christian Hassager was supported by an unrestricted grant from Lundbeck Foundation (R186‐2015‐2132), the Danish Heart Foundation, and Novo Nordisk Foundation (NNF20OC0064043), and has received speaker fees from Abiomed, BD, and Novo Nordisk outside the submitted work. Jacob E. Møller reports Institutional research grant from Abiomed and Novo Nordic Foundation, serves on advisory board for Boston Scientific and Magenta, and has received speaker fees from Abbott and Abiomed; all outside the submitted work. The other authors declare no conflicts of interest.

## Data Availability

Data sharing not applicable to this article as no datasets were generated or analyzed during the current study.
